# TBK1 inhibitor amlexanox exerts anti-cancer effects against endometrial cancer by regulating AKT/NF-κB signaling

**DOI:** 10.7150/ijbs.100212

**Published:** 2025-01-01

**Authors:** Jiha Shin, Jihoon Lim, Daewon Han, Solji Lee, Nak Song Sung, Jong-Seok Kim, Do Kyung Kim, Hoi Young Lee, Sung Ki Lee, Jongdae Shin, Jeong Sig Kim, Hwan-Woo Park

**Affiliations:** 1 Department of Cell Biology, Konyang University College of Medicine, Daejeon 35365, Republic of Korea.; 2 Department of Surgery, Konyang University Hospital, Daejeon 35365, Republic of Korea.; 3 Myunggok Medical Research Institute, Konyang University College of Medicine, Daejeon 35365, Republic of Korea.; 4 Department of Anatomy, Konyang University College of Medicine, Daejeon 35365, Republic of Korea.; 5 Department of Pharmacology, Konyang University College of Medicine, Daejeon 35365, Republic of Korea.; 6 Department of Obstetrics and Gynecology, Konyang University Hospital, Daejeon, Republic of Korea.; 7 Department of Obstetrics and Gynecology, Soonchunhyang University Seoul Hospital, Seoul 04401, Republic of Korea.

**Keywords:** TBK1, endometrial cancer, xenograft, shRNA, prognosis, NF-κB

## Abstract

Endometrial cancer, a common gynecological malignancy, poses significant clinical challenges, particularly in advanced or recurrent cases. TANK-binding kinase 1 (TBK1), a serine/threonine kinase, plays crucial roles in inflammation and immunity by activating nuclear factor (NF)-κB and interferon regulatory factor 3. However, its specific roles in endometrial cancer remain unknown. In this study, we aimed to investigate the anti-cancer effects and underlying mechanisms of amlexanox, a TBK1 inhibitor, against endometrial cancer. The main genetic mutations in TBK1 were found to be mRNA downregulation and missense mutations. Kaplan-Meier plotter analysis revealed that low TBK1 expression was associated with a good prognosis in patients with uterine corpus endometrial carcinoma (UCEC). *In vitro* experiments demonstrated that *TBK1* knockdown or amlexanox significantly inhibited the proliferation, cell cycle progression, and migration of endometrial cancer cells. Furthermore, the inhibitory effects of targeting TBK1 on cancer cell proliferation and migration were mediated by the protein kinase B (AKT)/NF-κB signaling pathway. Xenograft experiments revealed that both amlexanox treatment and *TBK1* knockdown effectively suppressed the tumor growth. Overall, this study highlights the potent anti-cancer effects of amlexanox against endometrial cancer by modulating AKT/NF-κB signaling, thus providing a new avenue for the development of novel TBK1-targeting therapeutic strategies for UCEC.

## Introduction

Endometrial cancer, the most common gynecological malignancy, poses a significant global health burden, with increasing incidence worldwide 1, 2. Despite advances in treatment, management of advanced or recurrent endometrial cancer remains a clinical challenge, necessitating the development of new therapeutic strategies 3, 4. Targeting specific signaling pathways involved in endometrial cancer progression has emerged as a promising therapeutic approach.

TANK-binding kinase 1 (TBK1), a serine/threonine kinase, plays important roles in various cellular processes, including inflammation and innate immunity, via activation of nuclear factor (NF)-κB and interferon regulatory factor 3 pathways 5-7. TBK1 is involved in malignant cell growth in different cancer types, including non-small cell lung cancer, breast cancer, and pancreatic ductal adenocarcinoma 8-11. Increased TBK1 expression and aberrant TBK1 activity promote cancer cell survival and proliferation 9, 12, 13. TBK1 is considered a potential therapeutic target owing to its crucial roles in various diseases, including autoimmune disorders, metabolic diseases, and cancer 6, 7, 14. Inhibition of TBK1 reduces the viability and proliferation of lung and thyroid cancer cells 15, 16. Although TBK1 is closely associated with cancer progression and cell viability, its specific roles in endometrial cancer remain unknown.

Recently, various TBK1-targeting inhibitors have been developed. These inhibitors have exhibited high efficacy in the treatment of TBK1-associated diseases 17. Amlexanox, a TBK1 inhibitor approved for the treatment of aphthous ulcers and asthma, inhibits obesity and type 2 diabetes 18-21. It has garnered interest owing to its potential anti-cancer effects 22, 23. NF-κB signaling pathway plays vital roles in cancer development and progression by affecting cell survival, proliferation, and inflammation 24, 25. In lung and thyroid cancer, TBK1 is a key regulator of the NF-κB and protein kinase B (AKT) signaling pathways 12, 16. Amlexanox shows potential for endometrial cancer targeting by targeting TBK1. However, its specific anti-cancer effects and mechanisms against endometrial cancer remain unclear.

In this study, we aimed to investigate the effects of amlexanox and TBK1 short hairpin (sh) RNA (shRNA) on the proliferation and migration of endometrial cancer cells by inhibiting TBK1 expression. Moreover, we conducted bioinformatic analyses of TBK1, including genetic alteration, survival, DNA methylation, copy number variation, and somatic cell mutation analyses, in patients with uterine corpus endometrial carcinoma (UCEC). We also explored the molecular mechanisms underlying the regulation of AKT/NF-κB signaling by TBK1 inhibition in endometrial cancer cells. Our findings provide valuable insights for the development of targeted therapies and clinical management of patients with endometrial cancer.

## Materials and Methods

### Reagents

Amlexanox, BX-795, and MK-2206 were purchased from Cayman Chemical Co. (Ann Arbor, MI, USA). Immunoblotting and immunostaining were performed using antibodies against phosphorylated (p)-TBK1, total TBK1, N-cadherin, E-cadherin, snail, vimentin, p-AKT, total AKT, p-NF-κB, total NF-κB, p-p70S6K, total p70S6K (Cell Signaling Technology, Danvers, MA, USA), α-tubulin, and β-actin (Developmental Studies Hybridoma Bank, Iowa City, IA, USA). Transforming growth factor (TGF)-β1 and insulin growth factor-1 were purchased from PeproTech Inc. (Rocky Hill, NJ, USA).

### Cell culture and treatment

HEC-1A (ATCC, USA) and Ishikawa human endometrial adenocarcinoma cell lines were cultured in the Dulbecco's modified Eagle's medium (DMEM; Welgene, Korea) supplemented with 10% fetal bovine serum (FBS; Welgene), 4.5 g/L D-glucose with L-glutamine, and 100 U/mL penicillin-streptomycin. Human embryonic kidney (HEK)-293T cells were cultured in DMEM supplemented with 10% FBS, 4.5 g/L D-glucose with L-glutamine, 110 mg/L sodium pyruvate, and 100 U/mL penicillin-streptomycin. Both cultures were maintained in a humidified 5% CO_2_ atmosphere at 37 °C. For TBK1 or AKT inhibitor treatment, HEC-1A and Ishikawa cells were incubated with amlexanox (50, 100, and 200 μM) or MK-2206 (25, 50, and 100 nM) for the indicated time points. The same volume of dimethyl sulfoxide was used as vehicle control.

### cBioPortal

cBioPortal (https://www.cbioportal.org/) is a comprehensive open resource for the exploration and visualization of large-scale cancer genomic databases, including The Cancer Genomic Atlas (TCGA), International Cancer Genome Consortium (ICGC), and Gene Expression Omnibus databases, which provide information on mutations, copy number alterations, and clinical correlations 26. We used cBioPortal to explore the mutation data of TBK1 in UCEC, evaluate the prognostic value of TBK1 in patients with UCEC, and determine the correlation between TBK1 methylation and gene expression levels.

### University of California Santa Cruz (UCSC) Xena

UCSC Xena (http://xena.ucsc.edu/) is a web-based resource for the visualization and analysis of cancer genomic databases, including TCGA, ICGC, TARGET, Genotype-Tissue Expression, and CCL 27. The UCSC Xena database was used to generate heat maps of TBK1 mRNA expression, methylation, copy number, and somatic mutations in patients with UCEC and controls. Data were obtained from TCGA-UCEC dataset with 606 samples.

### Tumor Immune Estimation Resource (TIMER) database analysis

TIMER2.0 (http://timer.cistrome.org/) is a web-based resource for the comprehensive analysis of gene expression and tumor-infiltrating immune cells in various cancer types. We used the Gene_Corr module of TIMER2.0 to analyze the correlation in expression levels between TBK1 and mechanistic target of rapamycin kinase and epithelial-mesenchymal transition (EMT) gene markers.

### Plasmids and viral transduction

Using a polyethylenimine reagent, HEK293T cells were transfected with the sh-luciferase (sh-Luc), sh-TBK1 (The RNAi Consortium, Broad Institute, Cambridge, MA, USA), GFP, and TBK1 (Addgene, Cambridge, MA, USA) constructs with packaging plasmids. Briefly, lentiviral supernatants were collected, filtered at 48 and 72 h after transfection, and concentrated using the Lenti-X concentrator (Takara, Japan), according to the manufacturer's protocol. Then, HEC-1A and Ishikawa cells were transduced with the concentrated lentiviruses in the presence of 4 μg/mL polybrene.

### Cell proliferation assay

HEC-1A and Ishikawa cells were seeded into 96-well plates at a density of 1 × 10^5^ cells/well with 100 μL of the respective culture medium. After allowing the cells to adhere overnight, they were treated with amlexanox and vehicle or infected with sh-TBK1 or sh-Luc. Cell proliferation was assessed via water-soluble tetrazolium salt (WST)-8 assay (Biomax, Korea), according to the manufacturer's instructions. WST-8 reagent was added to each well and incubated for 30 min at 37 °C. Absorbance was measured at 450 nm using the Epoch 2 microplate reader (BioTek Instruments, Winooski, VT, USA).

### BrdU incorporation assay

Cells were incubated with 10 μM 5-bromo-2'-deoxyuridine (BrdU) for 4 h. Following incubation, the cells were washed with PBS and fixed with 4% paraformaldehyde (pH 7.4, Biosesang, Seongnam, Korea). DNA denaturation was then achieved by treating the cells with 2N HCl. The incorporated BrdU was detected using an anti-BrdU antibody (Invitrogen, Carlsbad, CA, USA), followed by incubation with an Alexa Fluor 488-conjugated secondary antibody (Thermo Fisher Scientific). Nuclei were counterstained with 4',6-diamidino-2-phenylindole (Thermo Fisher Scientific). The labeling index was expressed as the proportion of BrdU-positive cells relative to the total cell count.

### Colony formation assay

HEC-1A and Ishikawa cells were seeded into 6-well plates at a density of 1,000 cells/well. After allowing the cells to attach overnight, they were cultured for 2 weeks in complete medium. The formed colonies were then fixed with a pre-chilled methanol for 10 min and stained with 0.5% crystal violet for 15 min. The number of colonies was counted from three independent experiments.

### Cell cycle assay

HEC-1A and Ishikawa cells were fixed with 70% cold ethanol for 30 min. For DNA staining, the fixed cells were washed and resuspended in phosphate-buffered saline (PBS) with propidium iodide (PI; 0.5 mg/mL) and RNase A (100 µg/mL) in the dark at 22-25 °C for 30 min. PI-stained cells were analyzed using the CytoFLEX S flow cytometer (Beckman-Coulter, USA).

### Reverse transcription-quantitative polymerase chain reaction (RT-qPCR)

Total RNA was extracted from xenograft tumor tissues, HEC-1A cells, and Ishikawa cells using TRIzol reagent (Takara), according to the manufacturer's instructions. It was reverse transcribed to cDNA using the cDNA synthesis kit (BioFact, Seoul, Korea). qPCR was performed using the QuantStudio 3 real-time PCR system (Life Technologies, Carlsbad, CA, USA) with the SYBR Green qPCR Master Mix (BioFact). The qPCR reaction was set up as follows: 10 μL of SYBR Green Master Mix, 1 μL of each primer (final concentration of 500 nM), 2 μL of cDNA template, and 6 μL of nuclease-free water in a total volume of 20 μL. The qPCR thermal cycling conditions were as follows: 5 min at 95 °C, 40 cycles of 15 s at 95 °C, 30 s at 60 °C, and 30 s at 72 °C, followed by a 5 min final extension at 72 °C. The relative expression of each target gene was analyzed using the comparative threshold cycle (Ct) method with an internal control gene to normalize the target gene expression level. All primers used in this study are listed in Table S1.

### Western blotting

Total protein was extracted from endometrial cancer tissues, HEC-1A cells, and Ishikawa cells using a radioimmunoprecipitation assay buffer with a protease inhibitor cocktail (Roche Diagnostics, Germany). Samples were homogenized and incubated on ice for 20 min, followed by centrifugation at 18,000 × *g* for 15 min at 4 °C. The total protein concentration was determined via bicinchoninic acid protein assay (Thermo Fisher Scientific, Waltham, MA, USA). Lysates were mixed with the sodium dodecyl sulfate Laemmli sample buffer, denatured by heating at 95 °C for 5 min, and separated via sodium dodecyl sulfate-polyacrylamide gel electrophoresis. Proteins were transferred onto a polyvinylidene fluoride membrane (Merck Millipore, Germany) using a semi-dry transfer system (Trans-Blot Turbo; Bio-Rad, Hercules, CA, USA). The transferred membranes were blocked with 5% non-fat milk in TBS-T for 1 h at room temperature. After blocking, the blots were incubated with primary antibodies against N-cadherin, E-cadherin, snail, vimentin, p-NF-κB, total NF-κB, p-AKT, total AKT, p-p70S6K, total p70S6K, β-actin, and α-tubulin overnight at 4 °C. After washing thrice with TBS-T for 10 min, the blots were incubated with the appropriate horseradish peroxidase-conjugated secondary antibodies for 1 h at 22-25 °C. After washing, the protein bands were visualized using the SuperSignal West Femto chemiluminescent substrate (Thermo Fisher Scientific) and detected using the Fusion Solo imaging system (Vilber Lourmat, France). ImageJ software (National Institutes of Health, Bethesda, MD, USA) was used to calculate the protein band intensities.

### Wound healing assay

HEC-1A and Ishikawa cells were seeded into 6-well plates and treated with amlexanox or TGF-β1 or transduced with sh-TBK1 or sh-Luc. When cells reached 90% confluency, a wound was created in the cell monolayer using a sterile 200-μL pipette tip to scratch a straight line across the well. Phase-contrast images of cell migration into the wound were captured at indicated time points using an inverted microscope (Ts2-FL; Nikon, Japan). The wound area at each time point was measured using the ImageJ software to determine the extent of wound closure.

### Transwell migration assay

HEC-1A and Ishikawa cells were treated with amlexanox or vehicle or transduced with sh-TBK1 or sh-Luc. After reaching 80-90% confluency, the cells were detached using trypsin-EDTA. Transwell inserts with 8-μm pores (BD Falcon, Durham, NC, USA) were inserted into 24-well plates. The cells were resuspended in a medium containing 1% FBS at a density of 5 × 10^5^ cells/mL. The cell suspension (200 μL) was added to the upper chamber of each transwell insert. The lower chamber was filled with a medium supplemented with 10% FBS to induce cell migration. After 24 h, non-migratory cells in the upper transwell chamber were removed with a cotton swab, and the migratory cells in the lower chamber were fixed with cold methanol and stained with crystal violet (Sigma-Aldrich, St. Louis, MO, USA). The average number of migrated cells was counted in five random fields at 100× magnification.

### Immunocytochemistry

HEC-1A and Ishikawa cells were fixed with 4% paraformaldehyde (pH 7.4) for 15 min at room temperature, washed thrice with PBS, and permeabilized with methanol at -20 °C. The cells were incubated for 1 h in a blocking solution with an anti-NF-κB antibody at 4 °C in a humidified chamber. After washing thrice with PBS, the cells were incubated with the Alexa Fluor 488-conjugated secondary antibody for 1.5 h at 22-25 °C. Nuclei were counterstained with 4',6-diamidino-2-phenylindole, and coverslips were mounted on slides using the ProLong Gold antifade reagent (Thermo Fisher Scientific). Fluorescent images were obtained using a laser scanning confocal microscope (LSM 700; Carl Zeiss, Germany).

### Establishment of a tumor xenograft model

All animal experiments were approved by the Institutional Animal Care and Use Committee of the Konyang University (approval no. P-23-30-A-01). Five-week-old female nude BALB/c mice (14-19 g, Nara Biotech, Korea) were housed under pathogen-free conditions with appropriate care and monitoring throughout the study period. HEC-1A cells (4 × 10^6^ cells/animal) were resuspended in 100 μL PBS and subcutaneously injected into the dorsal flank of the mice. When average tumor volume reached approximately 50 mm^3^ (day 0), tumor-bearing mice were randomly divided into two groups (n = 6) and intraperitoneally administered with amlexanox (5 mg/kg) or vehicle containing 5% Tween 80 daily for two weeks. To establish sh-Luc and sh-TBK1 tumor models, five-week-old female BALB/c nude mice were randomly divided into two groups (n = 6). Lentiviral sh-Luc or sh-TBK1-infected HEC-1A cells (4 × 10^6^ cells/animal) were resuspended in 100 μL PBS and subcutaneously injected into the dorsal flank of the mice. After one week, tumor growth was monitored every three days using linear calipers. The tumor volume was calculated using the following formula: Volume = length × width^2^ × 0.52 (mm^3^). Mice were sacrificed via CO_2_ asphyxiation 4-5 weeks after tumor implantation. Then, their tissues were excised, weighed, and used for subsequent analysis.

### Statistical analyses

Results are presented as the mean ± standard error of the mean. Unless otherwise indicated, data shown in the figures represent at least three independent experiments. Statistical analysis of data of the two groups was conducted using a two-tailed Student's *t*-test. Multiple groups were compared using one-way analysis of variance with Tukey's post-hoc test. Statistical significance was set at p < 0.05.

## Results

### *TBK1* genetic mutations and their associations with the overall survival (OS) and disease-free survival (DFS) of patients with UCEC

**Figure 1:**
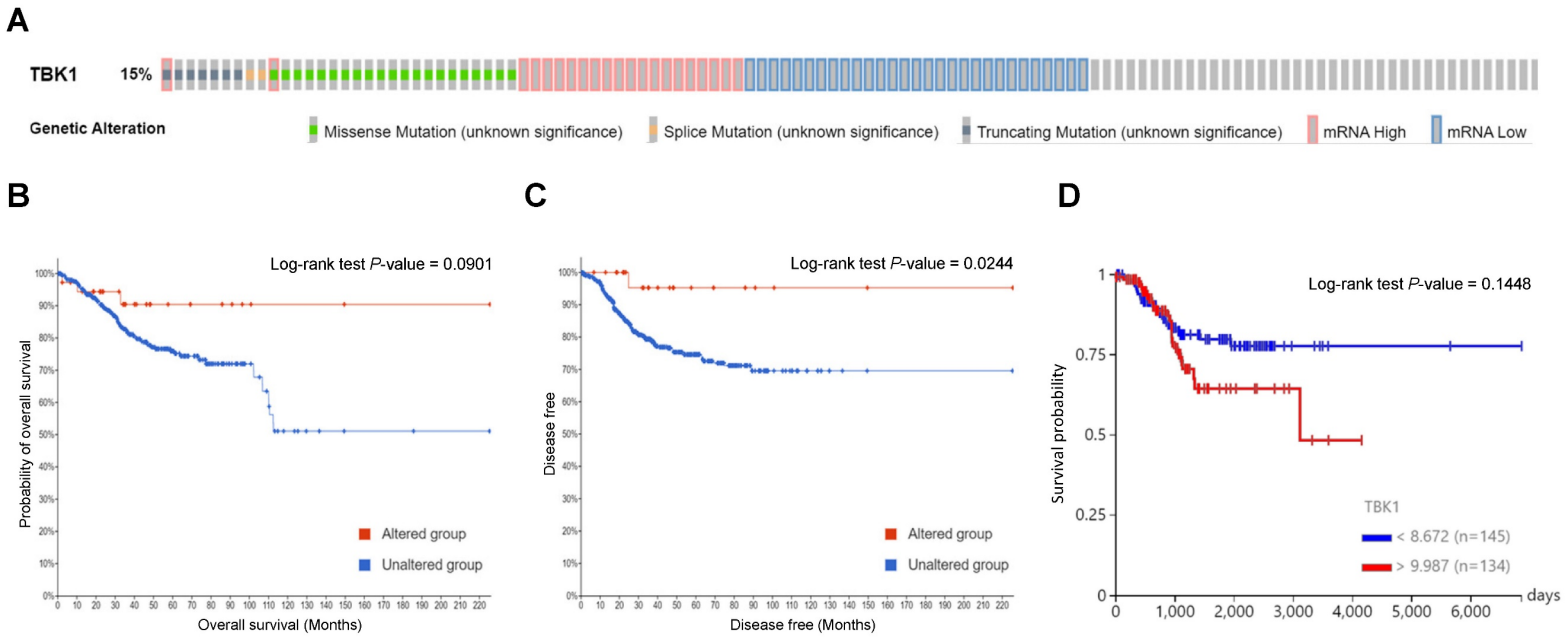
Genetic mutations in TANK-binding kinase 1 (*TBK1*) and their association with endometrial cancer prognosis. (A) Oncoprint analysis revealed the proportion and distribution of samples with different types of alterations in TBK1. (B and C) Kaplan-Meier curves of the overall and disease-free survival of patients with endometrial cancer in *TBK1*-altered and *TBK1*-unaltered groups. (D) Kaplan-Meier curves of overall survival according to TBK1 expression level in patients with endometrial cancer.

To determine the mutation characteristics of TBK1 and their correlation with patient survival in UCEC, we used the c-BioPortal database. TBK1 exhibited a high mutation frequency of 15% in UCEC on TCGA and PanCancer Atlas. The main genetic mutations in TBK1 were mRNA downregulation and missense mutations (Fig. 1A). Kaplan-Meier plots and log-rank tests revealed that aberrant expression of *TBK1* mRNA was significantly associated with high OS (Fig. 1B) and DFS (Fig. 1C) in patients with UCEC. Furthermore, low TBK1 expression was associated with a better prognosis than those with high TBK1 expression in patients with UCEC (Fig. 1D). Subsequently, associations between TBK1 expression levels and copy number variance, somatic mutations, and DNA methylation were analyzed. As shown in Fig. S1A, no obvious alternations in the copy numbers, somatic mutations, and DNA methylation were observed that contributed to the TBK1 expression. Correlation analysis revealed that two TBK1 CpG sites (cg04466273 and cg21722680) were not significantly associated with TBK1 expression in patients with UCEC (Fig. S1B and C).

### *TBK1* deficiency suppressed proliferation and cell cycle progression in endometrial cancer cells

**Figure 2:**
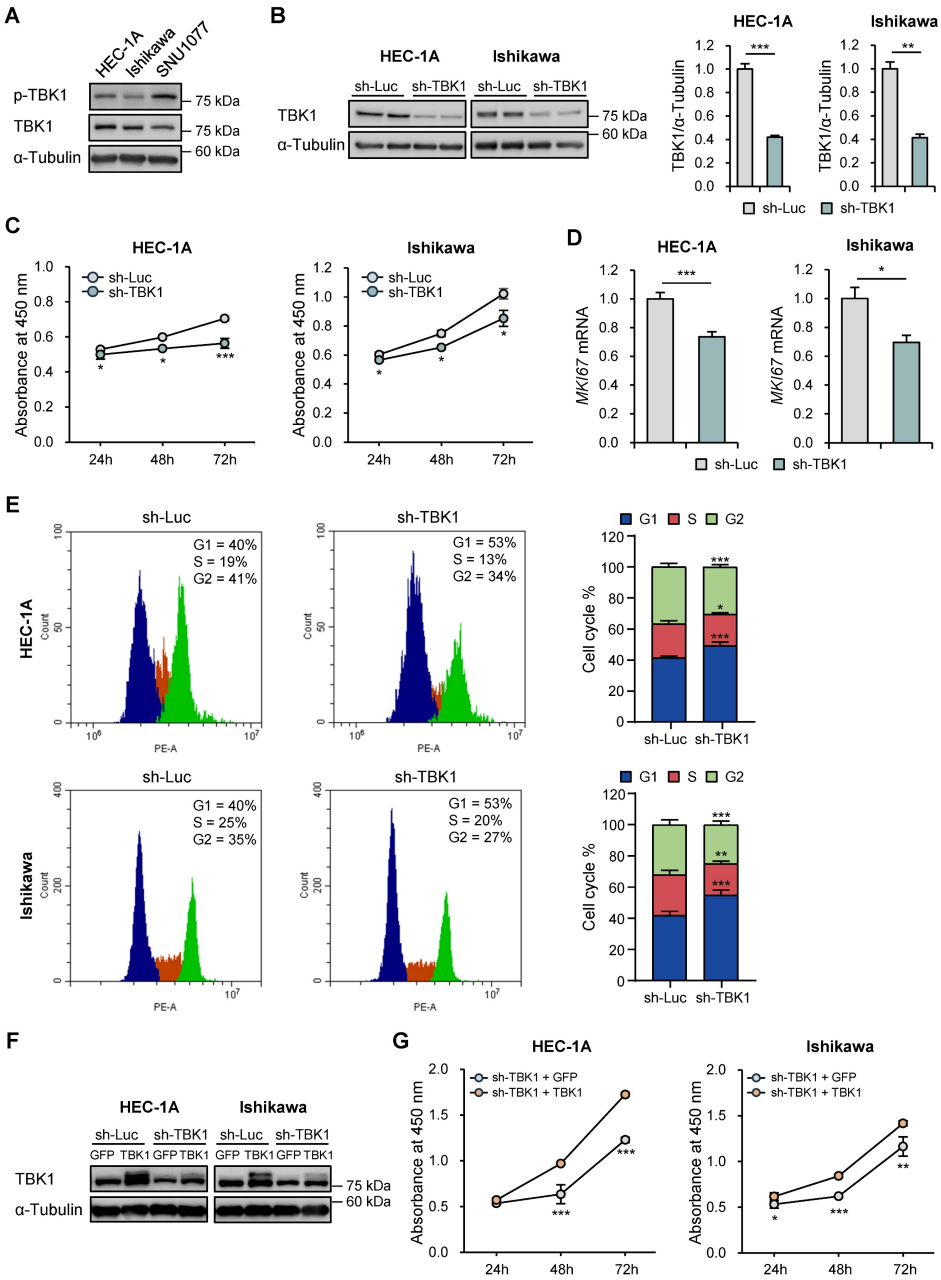
*TBK1* knockdown suppresses proliferation and promotes cell cycle arrest in endometrial cancer cells. (A) Immunoblots of p-TBK1 and TBK1 in the cell lysates of three endometrial cancer cell lines. (B) Immunoblots of TBK1 in the cell lysates of lentiviral sh-TBK1- or sh-Luc-infected HEC-1A and Ishikawa cells. Band intensities were quantified and normalized to the control levels. (C) Water-soluble tetrazolium salt (WST)-8 assay of HEC-1A and Ishikawa cells infected with sh-TBK1 or sh-Luc lentiviruses at 24 h, 48 h, and 72 h. (D) Reverse transcription-quantitative polymerase chain reaction (RT-qPCR) analysis of *MKI67* mRNA levels in HEC-1A and Ishikawa cells infected with sh-TBK1 or sh-Luc lentiviruses. (E) Cell cycle analysis of HEC-1A and Ishikawa cells infected with sh-TBK1 or sh-Luc lentiviruses via propidium iodide (PI) staining followed by flow cytometry. (F) Immunoblots of TBK1 in the cell lysates of TBK1 knockdown cells transfected with GFP or TBK1-Flag. (G) WST-8 assay of TBK1 knockdown cells transfected with GFP or TBK1-Flag. Data are represented as the mean ± standard error of the mean (SEM). *p < 0.05, **p < 0.01, and ***p < 0.001.

We examined the expression of phosphorylated and total TBK1 in the three endometrial cancer cell lines. The phosphorylated and total forms of endogenous TBK1 were expressed in all three cell lines. The phosphorylated form showed the highest expression in HEC-1A and SNU-1077 cells, whereas the total form showed the highest expression in HEC-1A and Ishikawa cells (Fig. 2A). Subsequently, *TBK1* was knocked down in HEC-1A and Ishikawa cells, and the knockdown was confirmed via western blotting (Fig. 2B). The effect of TBK1 on endometrial cancer cell proliferation was investigated using the WST-8 assay. *TBK1* knockdown significantly decreased the proliferation of HEC-1A and Ishikawa cells compared to that of the control cells (Fig. 2C). We further confirmed the inhibitory effect of *TBK1* knockdown on the expression levels of the proliferation marker, MKI67, in HEC-1A and Ishikawa cells (Fig. 2D). Next, we investigated the effect of TBK1 on cell cycle progression. Flow cytometry revealed that *TBK1* knockdown significantly increased the percentage of cells in G1 phase and decreased the percentage of cells in S phase of the cell cycle (Fig. 2E). Therefore, *TBK1* deficiency induced G1 cell cycle arrest in endometrial cancer cells. Next, gene expression levels of the cyclin-dependent kinase (CDK) inhibitors, *CDKN1A* (encoding p21) and *CDKN1B* (encoding p27), were determined via RT-qPCR. *TBK1* knockdown significantly increased the expression levels of *CDKN1A* and *CDKN1B* (Fig. S2). These results suggest that *TBK1* knockdown regulates the cell cycle-related gene expression in endometrial cancer cells.

To determine whether restoring TBK1 expression could reverse the effects of TBK1 knockdown on endometrial cancer cell proliferation, we reintroduced TBK1 into the TBK1 knockdown cells using a TBK1 expression vector. Immunoblotting analysis confirmed that TBK1 expression was restored in HEC-1A and Ishikawa cells (Fig. 2F). The WST-8 assay showed that overexpression of TBK1 rescued the proliferation defect induced by TBK1 knockdown (Fig. 2G).

### Amlexanox inhibited proliferation and survival in endometrial cancer cells

**Figure 3:**
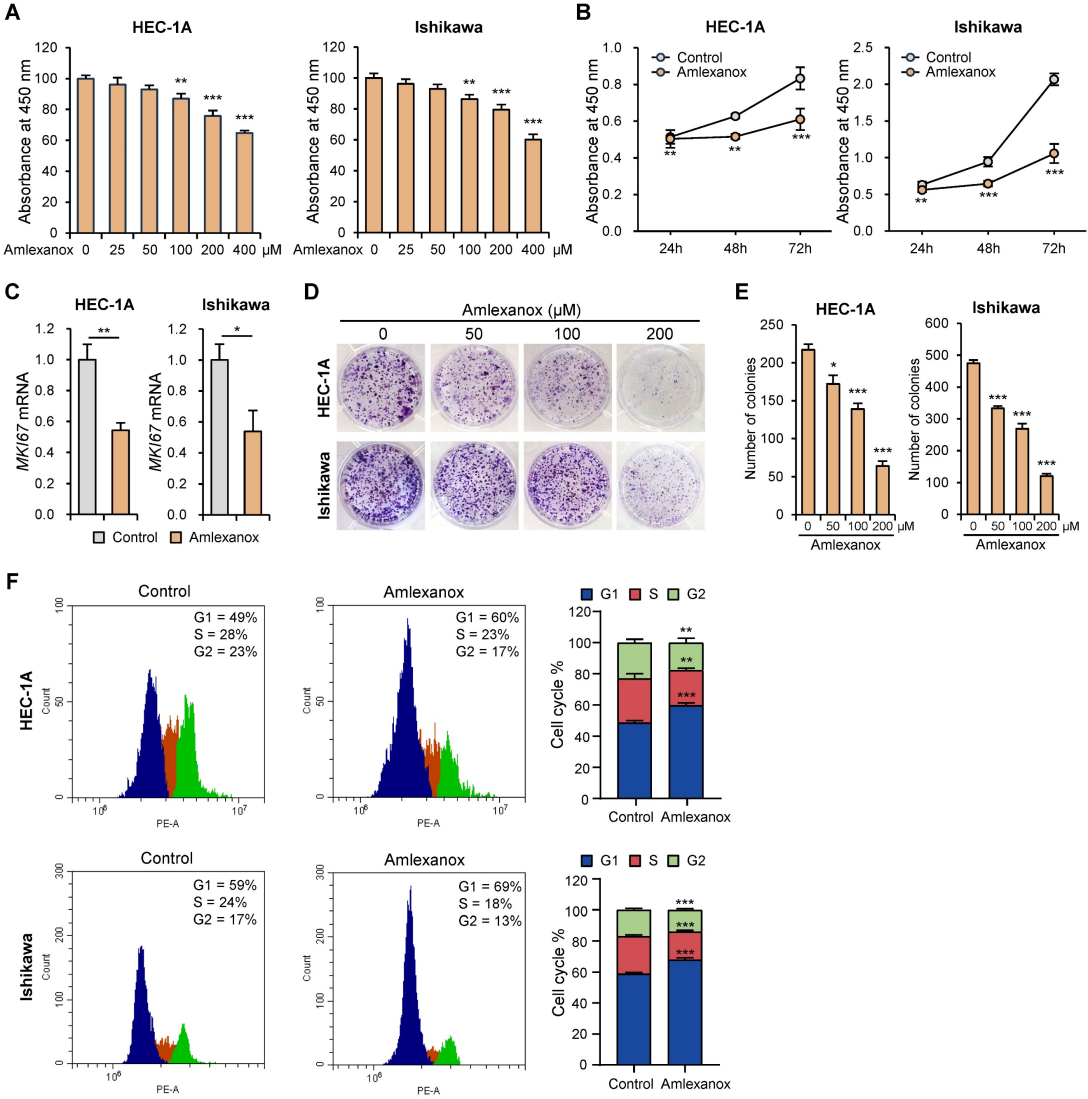
Amlexanox suppresses proliferation and promotes cell cycle arrest in endometrial cancer cells. (A) WST-8 assay of HEC-1A and Ishikawa cells treated with the indicated concentrations of amlexanox (25-400 μM) for 24 h. (B) WST-8 assay of HEC-1A and Ishikawa cells treated with 100 μM amlexanox for 24 h, 48 h, and 72 h. (C) RT-qPCR analysis of *MKI67* mRNA levels in HEC-1A and Ishikawa cells treated with 100 μM amlexanox for 24 h. (D) Colony formation assays of HEC-1A and Ishikawa cells treated with the indicated concentrations of amlexanox (50-200 μM). (E) Quantification of the colony formation assay shown in (D). (F) Cell cycle analysis of HEC-1A and Ishikawa cells treated with 100 μM amlexanox for 24 h via PI staining followed by flow cytometry. Data are represented as the mean ± SEM. *p < 0.05, **p < 0.01, and ***p < 0.001.

To determine the effect of amlexanox on endometrial cancer cell growth, we assessed cell proliferation using the WST-8 assay. Amlexanox suppressed the proliferation of HEC-1A and Ishikawa cells in a dose-dependent manner (Fig. 3A). It also significantly decreased the proliferation of HEC-1A and Ishikawa cells in a time-dependent manner (Fig. 3B). Next, we examined the effect of amlexanox on MKI67 expression. Amlexanox markedly decreased *MKI67* mRNA levels in HEC-1A and Ishikawa cells (Fig. 3C). BrdU incorporation assay showed a significant reduction in DNA synthesis in amlexanox-treated cells compared to control cells (Fig. S3A and S3B). We also conducted a colony formation assay to evaluate the long-term effects of amlexanox on cell survival and proliferation. The results showed a marked decrease in both the number and size of colonies formed by cells treated with amlexanox (Fig. 3D and E), suggesting a substantial inhibition of cell proliferation and clonogenic potential. To determine whether amlexanox affects cell cycle progression, we investigated the cell cycle phases using flow cytometry. Consistent with the inhibitory effect of *TBK1* knockdown on cell cycle, the G1 phase percentages of HEC-1A and Ishikawa cells were significantly higher in amlexanox-treated cells than in the control cells (Fig. 3F). Subsequently, we measured the expression levels of cell cycle-related genes. Amlexanox increased the expression of *CDKN1A* and *CDKN1B* in HEC-1A and Ishikawa cells (Fig. S3C). Immunoblotting analysis revealed that amlexanox increased the levels of cleaved PARP in a dose-dependent manner (Fig. S3D). Collectively, these data suggest that amlexanox suppresses proliferation and promotes cell cycle arrest and apoptosis in endometrial cancer cells.

### Downregulation of TBK1 expression was associated with EMT and migration in endometrial cancer cells

**Figure 4:**
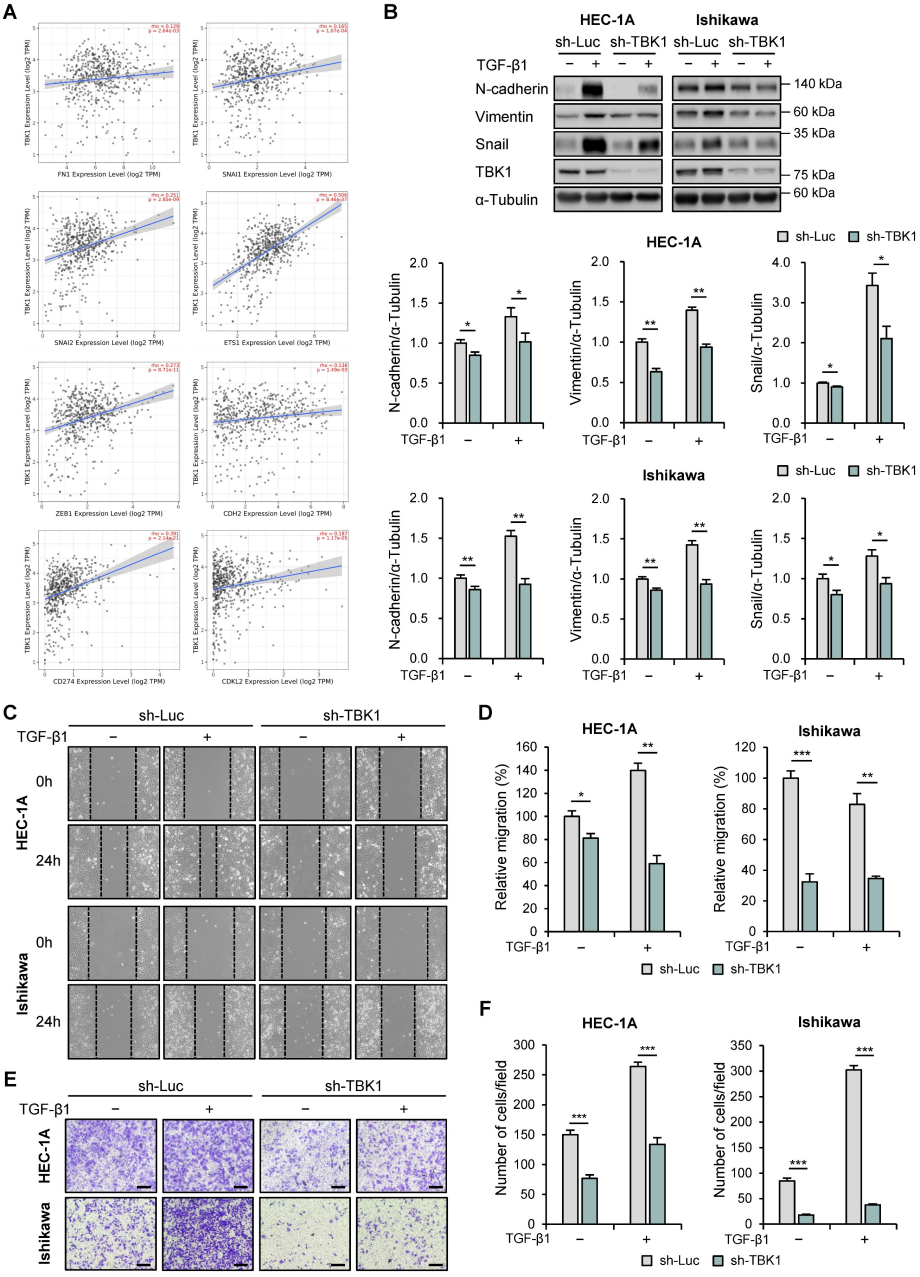
TBK1 expression is correlated with the epithelial-mesenchymal transition (EMT)-related marker expression and cell migration in endometrial cancer. (A) Spearman correlation analysis between TBK1 and EMT-related gene expression in The Cancer Genomic Atlas (TCGA) endometrial cancer database. (B) Immunoblots of N-cadherin, vimentin, snail, and TBK1 in the cell lysates of lentiviral sh-TBK1- or sh-Luc-infected HEC-1A and Ishikawa cells treated with 10 ng/mL transforming growth factor (TGF)-β1 or vehicle. Band intensities were quantified and normalized to the control levels. (C) Wound-healing assay of lentiviral sh-TBK1- or sh-Luc-infected HEC-1A and Ishikawa cells treated with 10 ng/mL TGF-β1 or vehicle. Images were taken 24 h after the scratch wound. (D) Quantification of cell migration expressed as a percentage of control values. (E) Transwell migration assay of lentiviral sh-TBK1- or sh-Luc-infected HEC-1A and Ishikawa cells treated with 10 ng/mL TGF-β1 or vehicle. Images were taken after 24 h under a light microscope. Scale bars, 200 μm. (F) Quantification of cell migration expressed as a percentage of control values. Data are represented as the mean ± SEM. *p < 0.05, **p < 0.01, and ***p < 0.001.

Next, we used the TIMER2.0 database to analyze the correlation between the expression levels of TBK1 and EMT-related genes using TCGA-UCEC data. *TBK1* expression levels were positively correlated with the expression levels of fibronectin 1 (*FN1*; r = 0.129, p = 2.64e-3), snail (*SNAI1*; r = 0.165, p = 1.07e-4), slug (*SNAI2*; r = 0.251, p = 2.85e-9), E26 transformation-specific (ETS) transcription factors 1 (*ETS1*; r = 0.506, p = 8.46e-37), zinc finger E-box binding homeobox 1 (*ZEB1*; r = 0.273, p = 8.71e-11), E-cadherin (*CDH1*; r = 0.136, p = 1.49e-3), PD-L1 (*CD274*; r = 0.391, p = 2.14e-21), and cyclin-dependent protein kinase-like 2 (*CDKL2*; r = 0.187, p = 1.17e-5) (Fig. 4A). We then determined the effects of *TBK1* knockdown on the expression levels of EMT-related proteins (N-cadherin, E-cadherin, vimentin, and snail) in HEC-1A and Ishikawa cells. Immunoblotting analysis revealed decreased levels of N-cadherin, vimentin, and snail in *TBK1*-knocked-down cells (Fig. 4B). However, *TBK1* knockdown did not affect the E-cadherin levels in HEC-1A and Ishikawa cells (Fig. S4). These results suggest that TBK1 is involved in EMT. We further investigated the metastasis of endometrial cancer cells following *TBK1* knockdown. Wound healing assay revealed that *TBK1* knockdown significantly reduced the migration of HEC-1A and Ishikawa cells compared to that of control cells (Fig. 4C and D). Transwell migration assay revealed that *TBK1* knockdown significantly inhibited the migration of endometrial cancer cells (Fig. 4E and F). To determine whether restoring TBK1 expression could reverse the effects of TBK1 knockdown on cell migration, we reintroduced TBK1 into TBK1 knockdown cells. Wound healing and transwell migration assays showed that overexpression of TBK1 significantly rescued the migration capability of the cells (Fig. S5A-D), indicating that TBK1 is crucial for maintaining the migratory properties associated with EMT.

### Amlexanox suppressed the expression of EMT-related markers and migration in endometrial cancer cells

**Figure 5:**
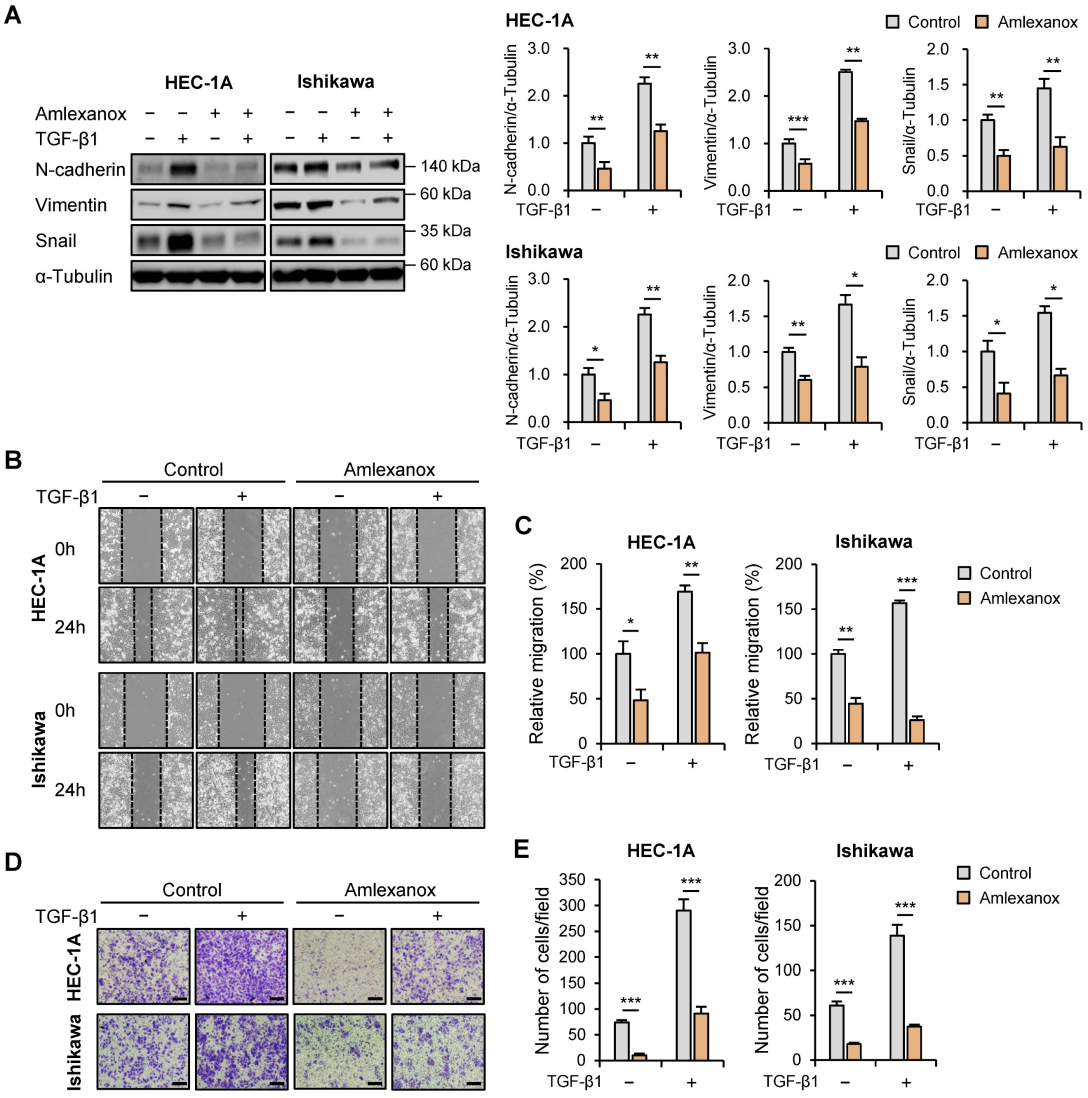
Amlexanox inhibits the migration of endometrial cancer cells. (A) Immunoblots of N-cadherin, vimentin, and snail in the cell lysates of HEC-1A and Ishikawa cells treated with 10 ng/mL TGF-β1 or 100 μM amlexanox for 36 h. Band intensities were quantified and normalized to the control levels. (B) Wound-healing assay of HEC-1A and Ishikawa cells treated with 10 ng/mL TGF-β1 or 100 μM amlexanox. Images were taken 24 h after the scratch wound. (C) Quantification of cell migration expressed as a percentage of control values. (D) Transwell migration assay of HEC-1A and Ishikawa cells treated with 10 ng/mL TGF-β1 or 100 μM amlexanox. Images were taken after 24 h under a light microscope. Scale bars, 200 μm. (E) Quantification of cell migration expressed as a percentage of control values. Data are represented as the mean ± SEM. *p < 0.05, **p < 0.01, and ***p < 0.001.

Subsequently, we investigated the effects of amlexanox on the expression levels of EMT-related proteins in HEC-1A and Ishikawa cells. Protein levels of N-cadherin, vimentin, and snail were significantly reduced in amlexanox-treated cells compared to those in control cells (Fig. 5A); however, E-cadherin expression levels were not affected (Fig. S6). We also examined the effects of BX795, another well-known TBK1 inhibitor, on the expression levels of EMT-related proteins. Similar to amlexanox, treatment with BX795 resulted in a significant decrease in the levels of N-cadherin and snail proteins (Fig. S7), suggesting that BX795 can effectively suppress EMT-related processes in these cells as well. We further evaluated the potential regulatory effect of amlexanox on endometrial cancer cell migration. Wound healing assay revealed that amlexanox markedly suppressed cell migration in both HEC-1A and Ishikawa cells (Fig. 5B and C). Transwell migration assay showed that the migration of HEC-1A and Ishikawa cells was significantly reduced after amlexanox treatment (Fig. 5D and E). Taken together, these data suggest that amlexanox suppresses EMT and migration of endometrial cancer cells.

### Targeting TBK1 inhibited the proliferation and migration of endometrial cancer cells via the AKT/NF-κB pathway

**Figure 6:**
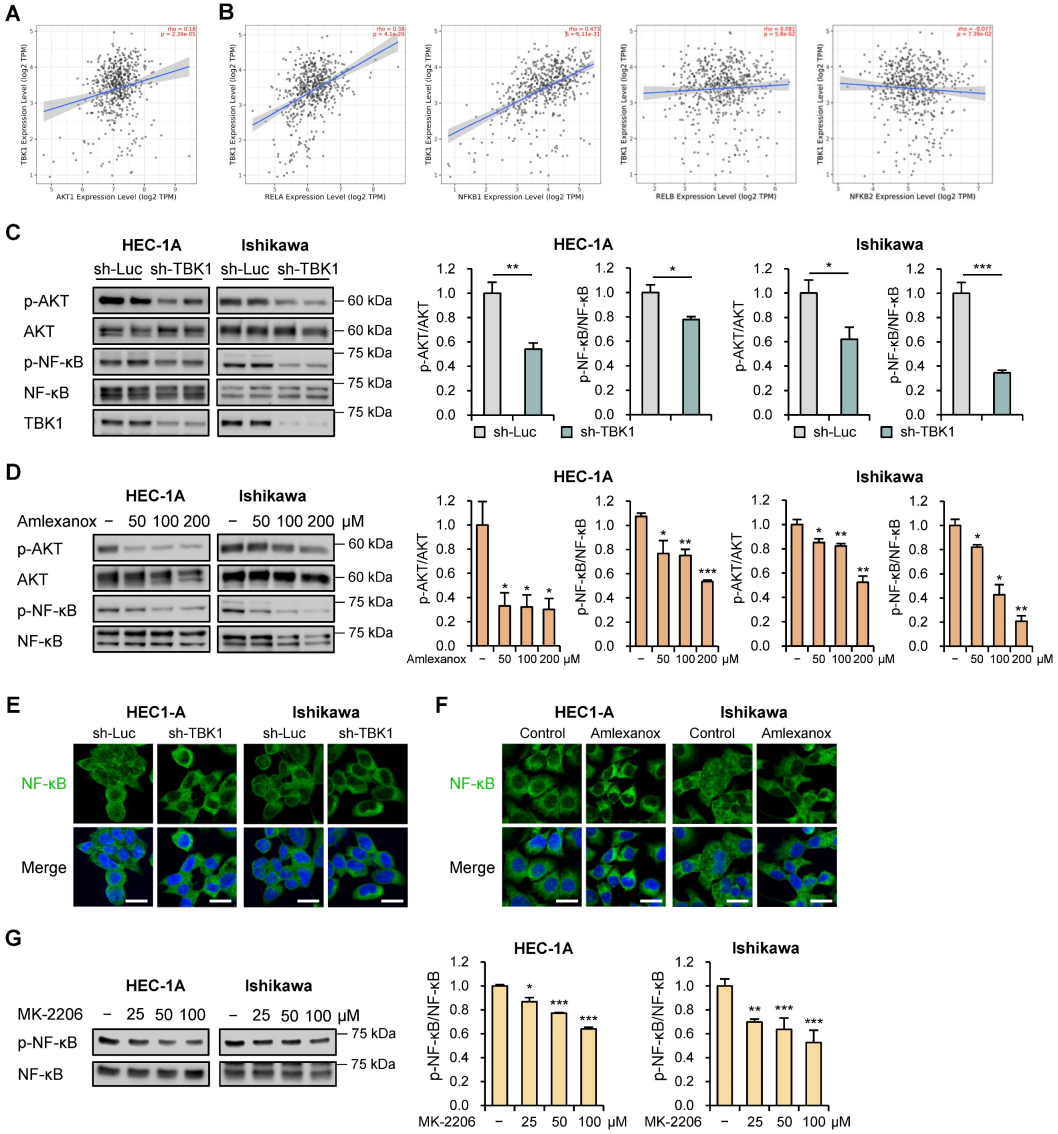
Targeting TBK1 attenuates nuclear factor (NF)-κB activation by inhibiting the protein kinase B (AKT) pathway in endometrial cancer cells. (A, B) Spearman correlation analysis between TBK1 and AKT- or NF-κB-related gene expression in TCGA endometrial cancer database. (C) Immunoblots of p-AKT, AKT, p-NF-κB, and NF-κB in the cell lysates of lentiviral sh-TBK1- or sh-Luc-infected HEC-1A and Ishikawa cells. (D) Immunoblots of p-AKT, AKT, p-NF-κB, and NF-κB in the cell lysates of HEC-1A and Ishikawa cells treated with amlexanox (50-200 μM) for 18 h. (E) Immunofluorescence staining for NF-κB in lentiviral sh-TBK1- or sh-Luc-infected HEC-1A and Ishikawa cells. (F) Immunofluorescence staining for NF-κB in HEC-1A and Ishikawa cells treated with 100 μM amlexanox for 24 h. Nuclei were stained with 4',6-diamidino-2-phenylindole (DAPI; blue). Scale bars, 20 μm. (G) Immunoblots of p-NF-κB and NF-κB in the cell lysates of HEC-1A and Ishikawa cells treated with MK-2206 (25-100 μM) for 4 h. Band intensities were quantified and normalized to the control levels. Data are represented as the mean ± SEM. *p < 0.05, **p < 0.01, and ***p < 0.001.

AKT/NF-κB signaling pathway contributes to tumor progression and invasion 28, 29. TBK1 directly interacts with and activates this pathway 30. To investigate their association, we evaluated the correlations between TBK1 and AKT/NF-κB pathway genes using the TIMER2.0 database. We observed strong correlations between *TBK1* and *AKT1* (r = 0.18, p = 2.39e-5), *RELA* (r = 0.38, p = 4.1e-20), and *NFKB1* (r = 0.473, p = 1.11e-31) levels in TCGA-UCEC data (Fig. 6A and B). However, no associations were observed between TBK1 levels and levels of non-canonical NF-κB subunit genes, *RELB* and *NFKB2* (Fig. 6B). Next, we explored whether AKT/NF-κB pathway is necessary for the anti-metastatic effects of targeting TBK1 against endometrial cancer cells. Immunoblotting analysis showed that the levels of p-AKT and p-NF-κB were significantly reduced by *TBK1* knockdown in HEC-1A and Ishikawa cells (Fig. 6C). Consistent with the inhibitory effect of *TBK1* knockdown on AKT/NF-κB pathway, amlexanox reduced the levels of p-AKT and p-NF-κB in a dose-dependent manner in cells (Fig. 6D). Moreover, *TBK1* knockdown and amlexanox treatment decreased the levels of p-NF-κB and NF-κB levels in the nuclei of both HEC-1A and Ishikawa cells, compared with those in the nuclei of control cells (Fig. 6E, F, S8A and S8B). We further assessed whether AKT inhibition affects NF-κB activity in endometrial cancer cells. AKT inhibitor MK-2206 significantly inhibited the phosphorylation of NF-κB in HEC-1A and Ishikawa cells (Fig. 6G). These findings suggest that targeting TBK1 decreases AKT phosphorylation and suppresses NF-κB pathway activation.

**Figure 7:**
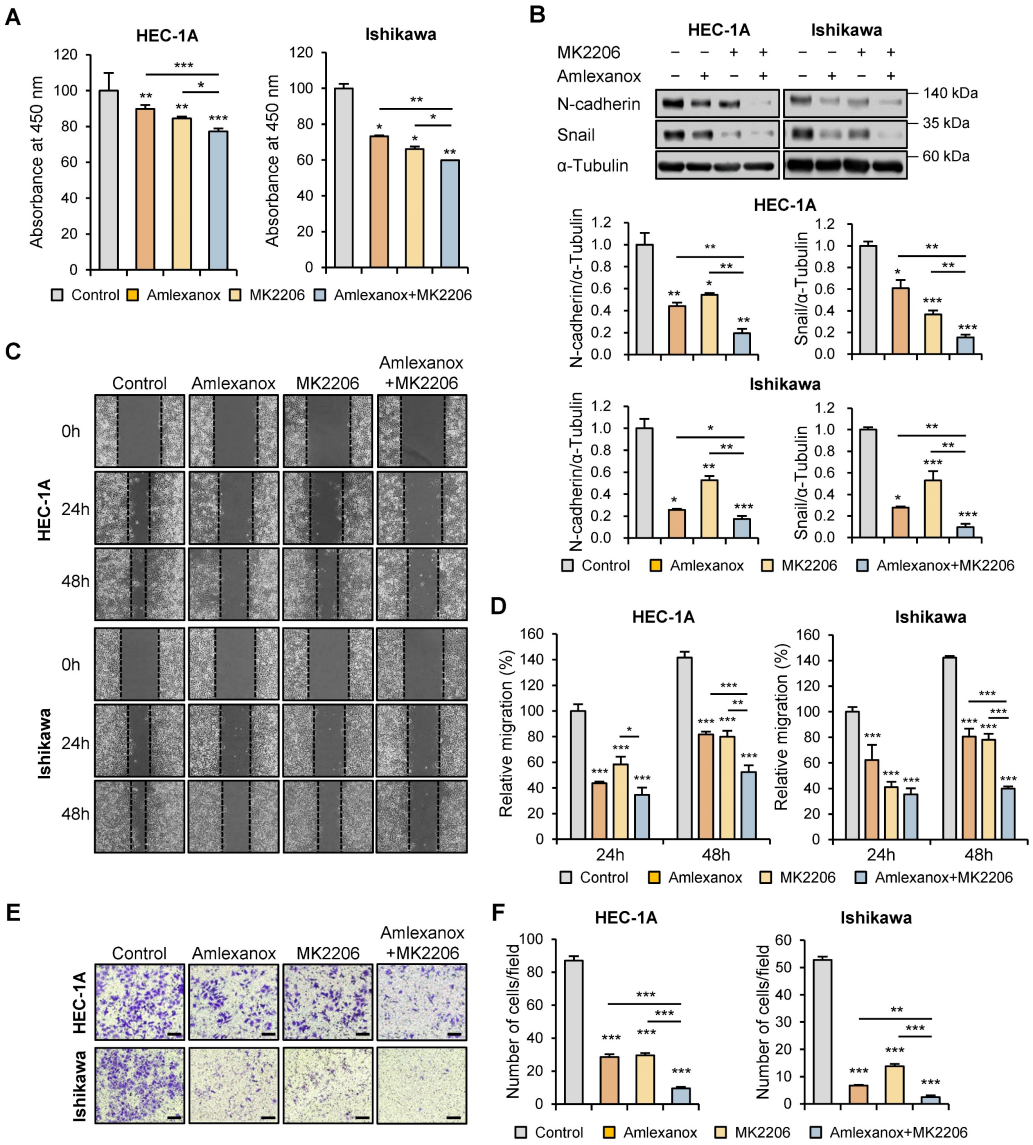
Targeting TBK1 inhibits the proliferation and migration of endometrial cancer cells via the AKT/NF-κB pathway. (A) WST-8 assay of HEC-1A and Ishikawa cells treated with 100 μM MK-2206 or 100 μM amlexanox for 24 h. (B) Immunoblots of N-cadherin and snail in the cell lysates of HEC-1A and Ishikawa cells pretreated with 100 μM MK-2206 or 100 μM amlexanox for 36 h in the presence of 10 ng/mL TGF-β1. Band intensities were quantified and normalized to the control levels. (C) Wound-healing assay of HEC-1A and Ishikawa cells pretreated with 100 μM MK-2206 or 100 μM amlexanox in the presence of 10 ng/mL TGF-β1. Images were taken 24 and 48 h after the scratch wound. (D) Quantification of cell migration expressed as a percentage of control values. (E) Transwell migration assay of HEC-1A and Ishikawa cells pretreated with 100 μM MK-2206 or 100 μM amlexanox in the presence of 10 ng/mL TGF-β1. Images were taken after 24 h under a light microscope. Scale bars, 200 μm. (F) Quantification of cell migration expressed as a percentage of control values. Data are represented as the mean ± SEM. *p < 0.05, **p < 0.01, and ***p < 0.001.

To further evaluate the regulatory effect of AKT inhibitor MK-2206 on endometrial cancer cell proliferation, WST-8 cell viability assay was performed using HEC-1A and Ishikawa cells after MK-2206 treatment. MK-2206 significantly suppressed cell proliferation, and this effect was enhanced by the combination of MK-2206 and amlexanox (Fig. 7A). Following this, we investigated whether AKT inhibition regulated the expression levels of EMT-related proteins in HEC-1A and Ishikawa cells. Immunoblotting analysis revealed that the protein levels of N-cadherin and snail were significantly reduced by MK-2206 and the combination of MK-2206 and amlexanox (Fig. 7B). Consistent with the inhibitory effects of the AKT inhibitor on the expression levels of EMT-related proteins, wound healing and transwell migration assays showed that MK-2206 suppressed the migration of both HEC-1A and Ishikawa cells (Fig. 7C-F). MK-2206 and amlexanox combination significantly reduced cell migration. These data suggest that the inhibitory effects of targeting TBK1 on cancer cell proliferation and migration are mediated by the AKT/NF-κB signaling pathway.

### Targeting TBK1 inhibited tumor growth *in vivo*

**Figure 8:**
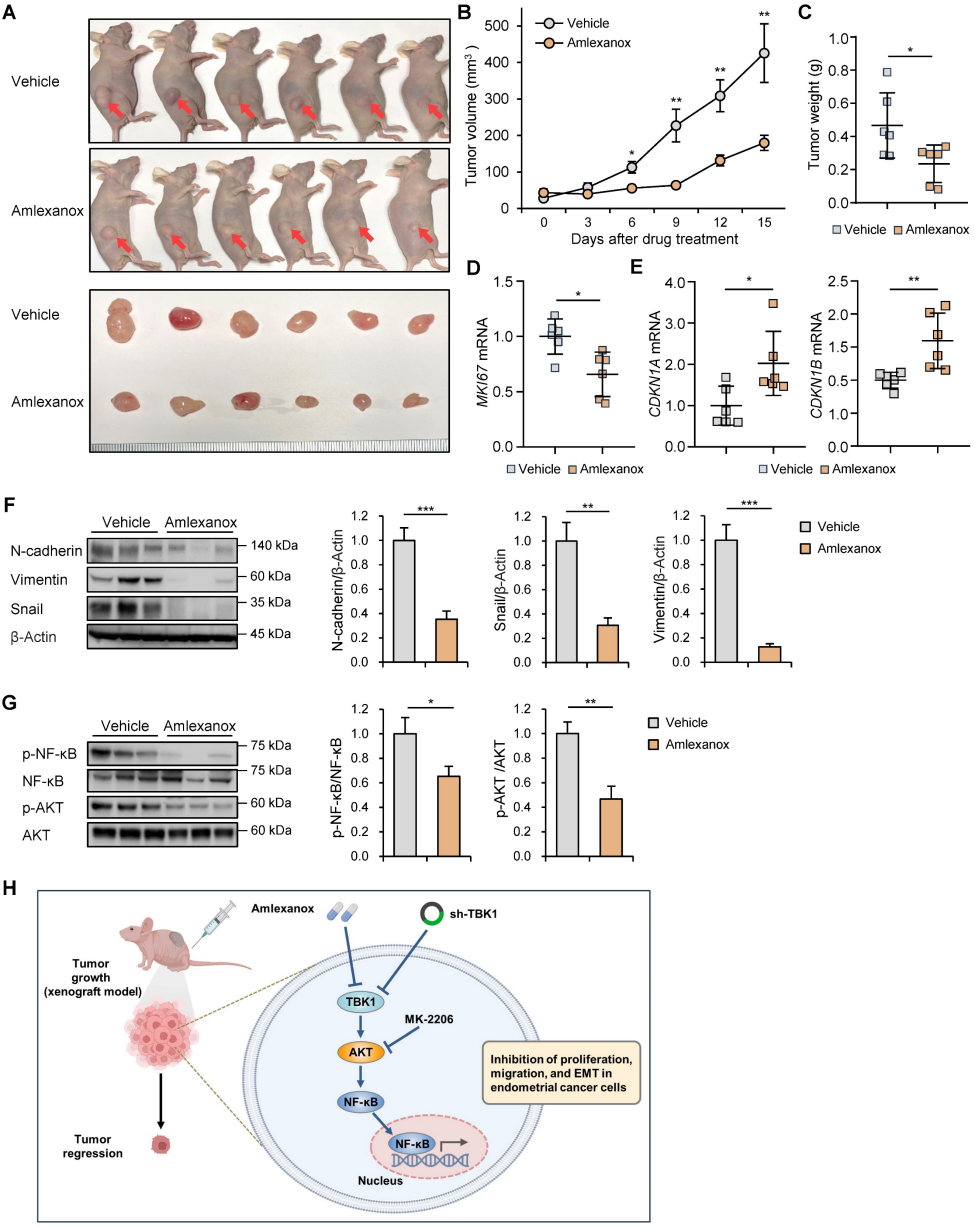
Targeting TBK1 inhibits tumor growth *in vivo*. (A) Representative images of subcutaneous xenograft tumors in nude mice treated with 5 mg/kg amlexanox or vehicle. Red arrows indicate the xenograft tumor. (B) Tumor growth curve in each group. (C) Tumor weights in each group. (D, E) RT-qPCR analysis of *MKI67, CDKN1A*, and* CDKN1B* mRNA levels in the xenograft tissues of each group. (F, G) Immunoblots of N-cadherin, vimentin, snail, p-AKT, AKT, p-NF-κB, and NF-κB in the xenograft tumor lysates of nude mice of each group. Band intensities were quantified and normalized to the control levels. Data are represented as the mean ± SEM. *p < 0.05, **p < 0.01, and ***p < 0.001. (H) Schematic diagram showing the anti-cancer mechanism of TBK1 inhibition, leading to decreased tumor growth, migration, and EMT in endometrial cancer cells.

To explore the roles of TBK1 in endometrial cancer-bearing mice, BALB/c nude mice were subcutaneously injected with lentiviral sh-TBK1- or sh-Luc-infected HEC-1A cells. Growth rate of xenograft tumors derived from the sh-TBK1 group was significantly lower than that of tumors derived from the sh-Luc group (Fig. S9A-C). To further verify the anti-tumor activity of amlexanox in xenograft models, HEC-1A cells were subcutaneously injected into BALB/c nude mice. The mice were intraperitoneally injected with amlexanox or vehicle for 15 d. Amlexanox significantly reduced the xenograft tumor growth in nude mice (Fig. 8A-C). Additionally, amlexanox inhibited MKI67 expression in xenograft tumors (Fig. 8D). Consistent with our results observed in HEC-1A and Ishikawa cells, RT-qPCR analysis showed that amlexanox treatment increased the expression of CDKN1A and CDKN1B in xenograft tumors (Fig. 8E). Immunoblotting analysis revealed that the expression levels of N-cadherin, vimentin, and snail were significantly decreased in the amlexanox-treated group compared with those in the control group (Fig. 8F). We further determined whether amlexanox treatment influenced AKT/NF-κB signaling in xenografts. Immunoblotting analysis revealed that phosphorylation of AKT and NF-κB was significantly lower in the amlexanox-treated group than in the control group (Fig. 8G). Collectively, these data suggest that targeting TBK1 inhibits the growth of endometrial cancer cells *in vivo*.

## Discussion

Endometrial cancer is a significant health concern worldwide, necessitating the exploration of new therapeutic strategies to improve patient outcomes 1. TBK1 is involved in various diseases, including metabolic diseases and cancer. 6, 7, 9, 12, 14 However, its specific roles in endometrial cancer remain unknown. In this study, we investigated the potential anti-cancer effects of amlexanox, a TBK1 inhibitor, against endometrial cancer and the mechanisms by which TBK1 regulates the proliferation and migration of endometrial cancer cells. We found that patients with UCEC with low TBK1 expression exhibited better prognosis than those with high TBK1 expression. Moreover, TBK1 expression was positively correlated with increased EMT-related marker expression in patients with UCEC. Treatment with a TBK1 inhibitor attenuated the growth of the endometrial cancer cells by inhibiting the AKT/NF-κB pathway (Fig. 8H). These findings are consistent with those of previous studies showing the beneficial effects of TBK inhibition on inflammatory and metabolic diseases and cancers 19, 22, 23, 31. The present study supports these findings by demonstrating the potential of TBK1 inhibition to suppress the growth of endometrial cancer cells.

Amlexanox, initially developed as an anti-inflammatory agent, has garnered increasing attention due to its potential therapeutic applications 6, 32. Our findings demonstrated that amlexanox effectively inhibited endometrial cancer cell proliferation and migration *in vitro*. These results are consistent with those of previous studies demonstrating the anti-cancer properties of amlexanox in non-small cell lung cancers, melanoma, and glioblastoma cells 22, 23, 33, 34. TBK1 is involved in the development and progression of multiple solid tumors. In lung and breast cancer cell lines, TBK1 phosphorylates Cdc20 at Ser134 and Cdh1 at multiple sites, including Thr20, Ser39, Ser42, Ser58, Ser131, and Ser151 12. This phosphorylation suggests that TBK1 modulates the activity of the anaphase-promoting complex/cyclosome, thereby influencing cell cycle dynamics and promoting cancer cell proliferation. Importantly, the finding that low TBK1 expression predicts a better prognosis in patients with UCEC highlights its potential as a therapeutic target and prognostic factor. Moreover, we found that inhibition of TBK1 through knockdown resulted in a marked reduction in proliferation and cell cycle progression of endometrial cancer cells. These findings are consistent with a growing body of evidence supporting the anti-cancer effects of TBK1 inhibition 8, 9. By disrupting critical signaling pathways, TBK1 inhibitors can effectively impair cancer cell survival and proliferation, thereby presenting a viable therapeutic strategy.

Our study demonstrated that amlexanox and TBK1 shRNA effectively suppressed the expression of EMT-related markers and reduced the migration capacity of endometrial cancer cells. This is particularly significant in the context of TGF-β signaling, which is known to play a dual role in cancer progression 35, 36. In early-stage cancer, TGF-β has been shown to exert tumor-suppressive effects by inducing apoptosis and cell cycle arrest. However, as the cancer progresses, TGF-β can switch roles and promote tumor progression and metastasis. In the present study, TGF-β treatment increased the expression of EMT-related markers and promoted the migration of endometrial cancer cells. However, when amlexanox or TBK1 knockdown were applied, we observed a significant reduction in these TGF-β-induced effects. These results suggest that TBK1 may be crucial in mediating the pro-tumorigenic effects of TGF-β, particularly in the context of EMT and cell migration.

AKT pathway, a key regulator of cell growth, survival, and metabolism, is commonly activated in various cancers and exhibits somatic mutations, copy number alterations, and aberrant epigenetic regulation in different cancer types 37, 38. Activated TBK1 induces AKT phosphorylation, thereby influencing the anti-apoptotic pathways and promoting cell survival 13, 30, 39. Herein, we observed a significant decrease in AKT phosphorylation after TBK1 inhibition, indicating its involvement in mediating the anti-cancer effects. Moreover, blocking AKT activation using MK-2206 suppressed the endometrial cancer cell proliferation and migration. This is consistent with previous reports that activation, mutation, and amplification of the AKT pathway contribute to cancer cell proliferation and survival 40, 41. AKT-mediated phosphorylation of downstream targets, including mTOR, GSK, and NF-κB, underscores its vital roles in driving the malignant phenotypes of cancer cells by promoting cell cycle progression, inhibiting apoptosis, and enhancing metabolic reprogramming 42-44. In this study, we found that amlexanox and TBK1 shRNA suppressed NF-κB phosphorylation in endometrial cancer cells. NF-κB is a transcription factor implicated in cancer-related inflammation, immune response, and progression. NF-κB activation leads to the transcription of various genes involved in inflammation, apoptosis, and metastasis, such as cytokines, Bcl-2 family proteins, and matrix metalloproteinases 45, 46. Dysregulated NF-κB activation promotes cancer cell survival, proliferation, and invasion, making it an attractive therapeutic target 45. By suppressing NF-κB signaling, TBK1 inhibition disrupts the pro-tumorigenic microenvironment and impedes cancer progression. Here, correlation between TBK1 inhibition by amlexanox and AKT/NF-κB axis suggests that the anti-cancer effects of amlexanox are associated with its ability to suppress the proliferation and migration of endometrial cancer cells via the AKT/NF-κB pathway.

While studies in other cancers have shown that TBK1 is a key regulator of these oncogenic pathways, there is a gap in the literature directly addressing the role of TBK1 in endometrial cancer. This study contributes to filling this gap by providing initial insights into how TBK1 might influence endometrial cancer progression through its interaction with the AKT/NF-κB pathway 13, 47, 48. The limited existing research underscores the need for further studies to explore the clinical relevance of TBK1 in endometrial cancer more comprehensively. This study highlights the potential of TBK1 inhibition as a therapeutic strategy for endometrial cancer. The ability of TBK1 inhibition to modulate multiple signals, including AKT and NF-κB, underscores its potential as a multi-target anti-cancer approach. Combination therapies concurrently targeting AKT and NF-κB signaling pathways may exhibit synergistic effects, amplifying the therapeutic efficacy of endometrial cancer treatment. However, further preclinical and clinical investigations are needed to validate the efficacy and safety of amlexanox as a therapeutic intervention for endometrial cancer. Moreover, mechanisms underlying the anti-cancer effects of TBK1 inhibition should be explored to facilitate its clinical translation.

In conclusion, our study revealed that TBK1 inhibition exerts anti-cancer effects against endometrial cancer by regulating the AKT/NF-κB pathway. Our findings suggest TBK1 inhibition as a promising therapeutic strategy for endometrial cancer, warranting further investigation for future translation into clinical practice.

## Supplementary Material

Supplementary figures and table.

## Abbreviations

TBK1: TANK-Binding Kinase 1
AKT: Protein Kinase B
NF-κB: Nuclear Factor Kappa B
UCEC: Uterine Corpus Endometrial Carcinoma
shRNA: Short Hairpin RNA
DMEM: Dulbecco's Modified Eagle Medium
FBS: Fetal Bovine Serum
HEK: Human Embryonic Kidney
TGF: Transforming Growth Factor
TCGA: The Cancer Genomic Atlas
ICGC: International Cancer Genome Consortium
UCSC: University of California, Santa Cruz
TIMER: Tumor Immune Estimation Resource
EMT: Epithelial-Mesenchymal Transition
sh-Luc: sh-luciferase
WST: Water-Soluble Tetrazolium Salt
PBS: Phosphate-Buffered Saline
PI: Propidium Iodide
RT-qPCR: Reverse Transcription-Quantitative Polymerase Chain Reaction
Ct: Comparative Threshold
DAPI: 4',6-diamidino-2-phenylindole
p: Phosphorylated
OS: Overall Survival
DFS: Disease-Free Survival
IRB: Institutional Review Board
SEM: Standard Error of the Mean
