# Supplementary material for: TBK1 inhibitor amlexanox exerts anti-cancer effects against endometrial cancer by regulating AKT/NF-κB signaling
Source: Int J Biol Sci. 2025 Jan 1;21(1):143–59. doi: 10.7150/ijbs.100212 (PMC11667804; doi:10.7150/ijbs.100212)
Supplement: Supplementary file 1 — Supplementary figures and table. [file ijbsv21p0143s1.pdf]

Supporting Information

Supplementary Table

Table S1. List of primer pairs used for RT-qPCR analysis.

Gene	Forward Primer (5`-3`)	Reverse Primer (5`-3`)
MKI67	ACGCCTGGTTACTATCAAAAG	CAGACCCATTTACTTGTGTTGGA
CDKN1A	TGTCCGTCAGAACCCATGC	AAAGTCGAAGTTCCATCGCTC
CDKN1B	AACGTGCGAGTGTCTAACGG	CCCTCTAGGGGTTTGTGATTCT
Cyclophilin	GCAAAGTGAAAGAAGGCA	CCATTCCTGGACCCAAAG

Supplementary Figure

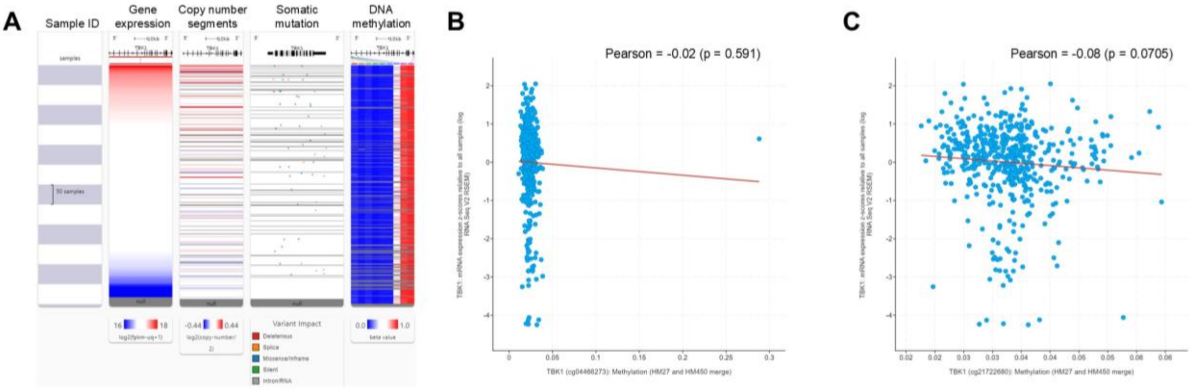

Figure S1. Analysis of TBK1 genetic and epigenetic alterations in patients with endometrial cancer. (A) Heat map of the expression levels, somatic mutations, copy number variants, and methylation status of TBK1 in patients with endometrial cancer. (B and C) Correlation analysis between methylation site (cg04466273 and cg21722680) and mRNA expression of TBK1 in endometrial cancer.

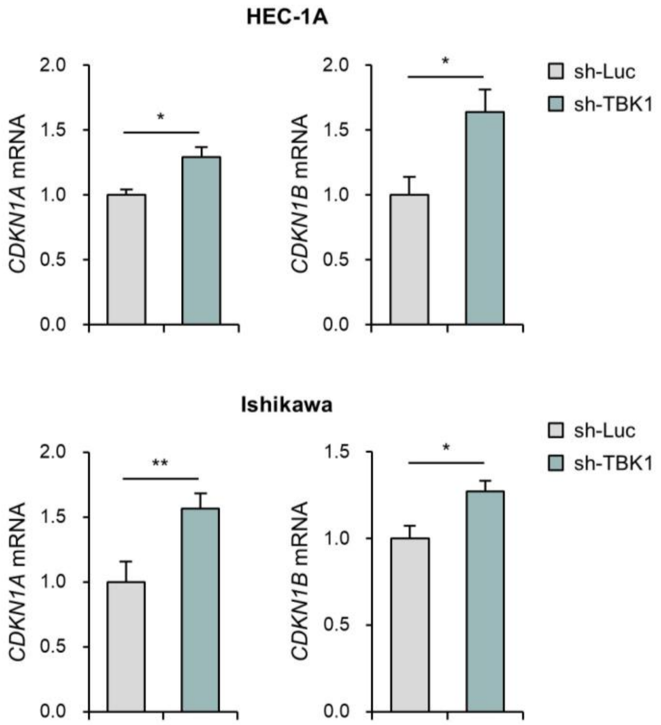

Figure S2. Reverse transcription-quantitative polymerase chain reaction (RT-qPCR) analysis of the mRNA levels of cyclin-dependent kinase inhibitors *CDKN1A* and *CDKN1B* in HEC-1A and Ishikawa cells infected with short hairpin RNA against TANK-binding kinase 1 (sh-TBK1) or luciferase (sh-Luc) lentiviruses. Data are represented as the mean ± standard error of the mean (SEM). *$p < 0.05$ and **$p < 0.01$.

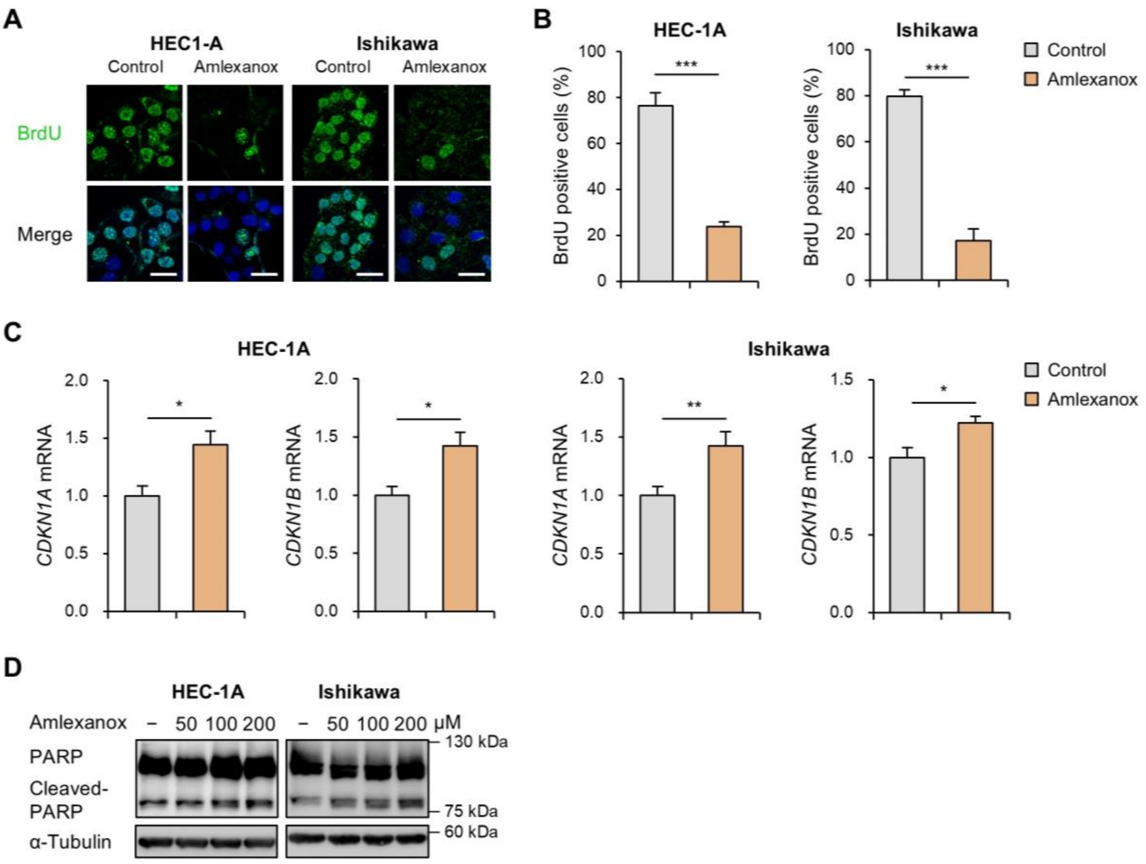

Figure S3. Amlexanox induces cell cycle arrest and apoptosis in endometrial cancer cells. (A) Immunofluorescence staining for BrdU in HEC-1A and Ishikawa cells treated with 100 µM amlexanox for 24 h. Nuclei were stained with 4',6-diamidino-2-phenylindole (DAPI; blue). Scale bars, 20 µm. (B) Quantification of BrdU positive cells. (C) RT-qPCR analysis of *CDKN1A* and *CDKN1B* mRNA levels in HEC-1A and Ishikawa cells treated with 100 µM amlexanox or vehicle for 24 h. Data are represented as the mean ± SEM. *p < 0.05 and **p < 0.01. (D) Immunoblots of PARP in the cell lysates of HEC-1A and Ishikawa cells treated with amlexanox (50–200 µM) for 24 h. Band intensities were quantified and normalized to the control levels.

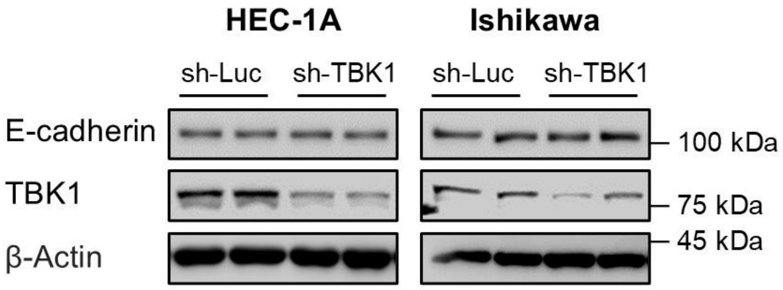

Figure S4. Effect of *TBK1* knockdown on E-cadherin expression in endometrial cancer cells. Immunoblots of E-cadherin in the cell lysates of lentiviral sh-TBK1- or sh-Luc-infected HEC-1A and Ishikawa cells.

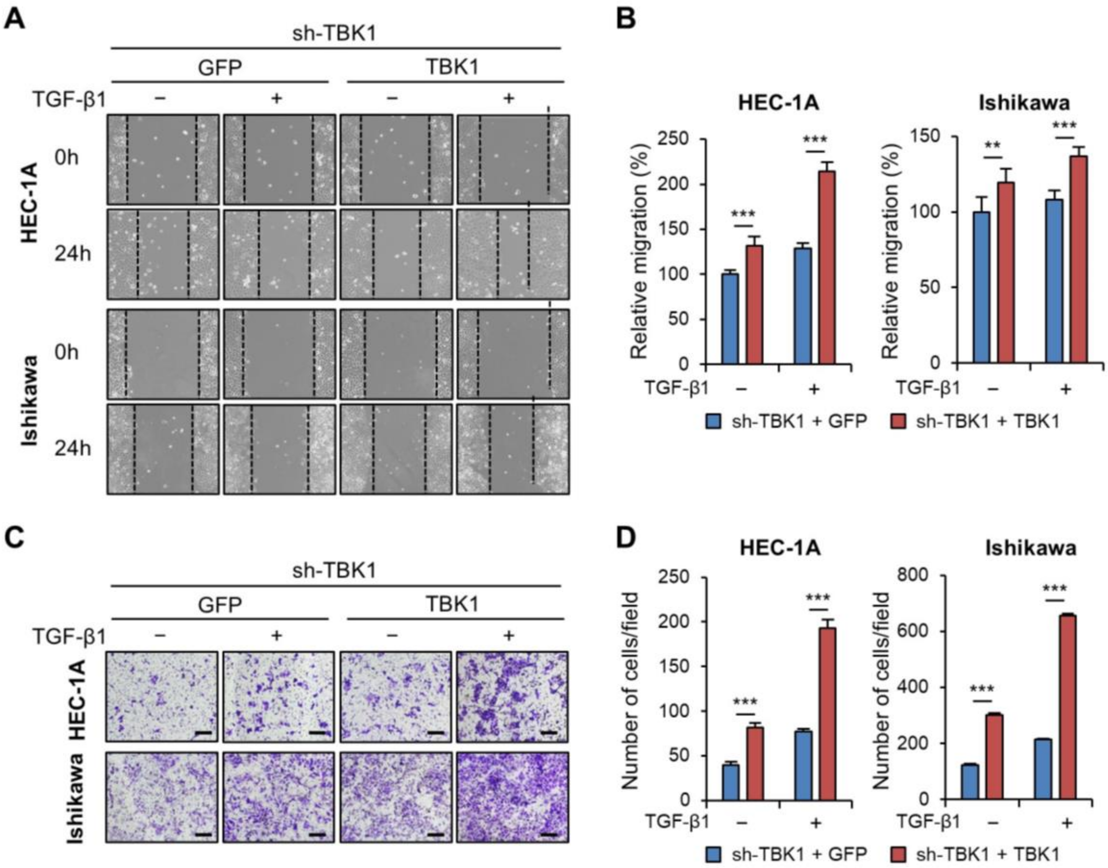

Figure S5. The rescue of TBK1 restores cell migration in TBK1 knockdown cells. (A) Wound-healing assay of TBK1 knockdown cells transduced with GFP or TBK1-Flag. Images were taken 24 h after the scratch wound. (B) Quantification of cell migration expressed as a percentage of control values. (C) Transwell migration assay of TBK1 knockdown cells transduced with GFP or TBK1-Flag. Images were taken after 24 h under a light microscope. Scale bars, 200 µm. (D) Quantification of cell migration expressed as a percentage of control values. Data are represented as the mean ± SEM. *p < 0.05, **p < 0.01, and ***p < 0.001.

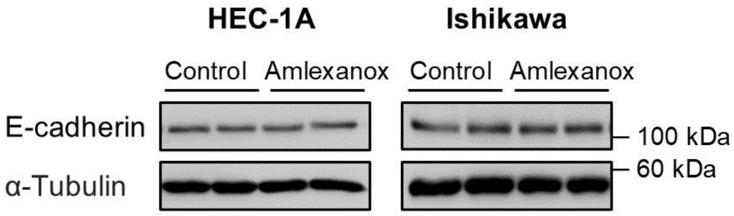

Figure S6. Effect of amlexanox on E-cadherin expression in endometrial cancer cells. Immunoblots of E-cadherin in the cell lysates of HEC-1A and Ishikawa cells treated with 100 μM amlexanox or vehicle for 24 h.

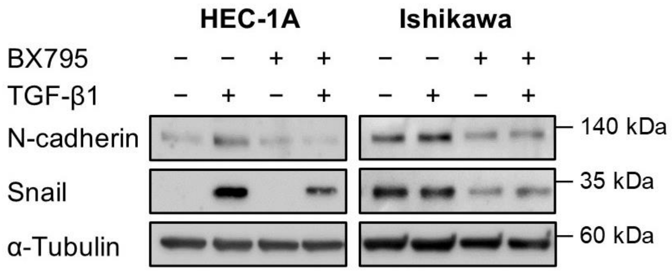

Figure S7. Effect of BX-795 on EMT-related protein expression in endometrial cancer cells. Immunoblots of N-cadherin and snail in the cell lysates of HEC-1A and Ishikawa cells treated with 5 μM BX-795 or vehicle for 24 h.

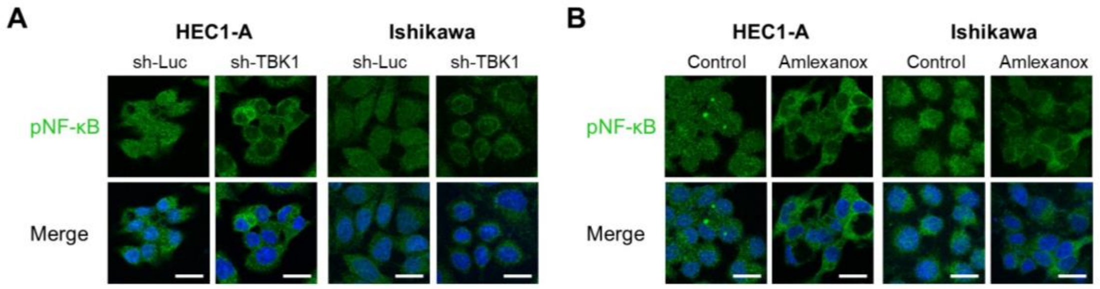

Figure S8. TBK1 regulates p-NF-κB localization in endometrial cancer cells. (A) Immunofluorescence staining for p-NF-κB in lentiviral sh-TBK1- or sh-Luc-infected HEC-1A and Ishikawa cells. (B) Immunofluorescence staining for p-NF-κB in HEC-1A and Ishikawa cells treated with 100 μM amlexanox for 24 h. Nuclei were stained with 4',6-diamidino-2-phenylindole (DAPI; blue). Scale bars, 20 μm.

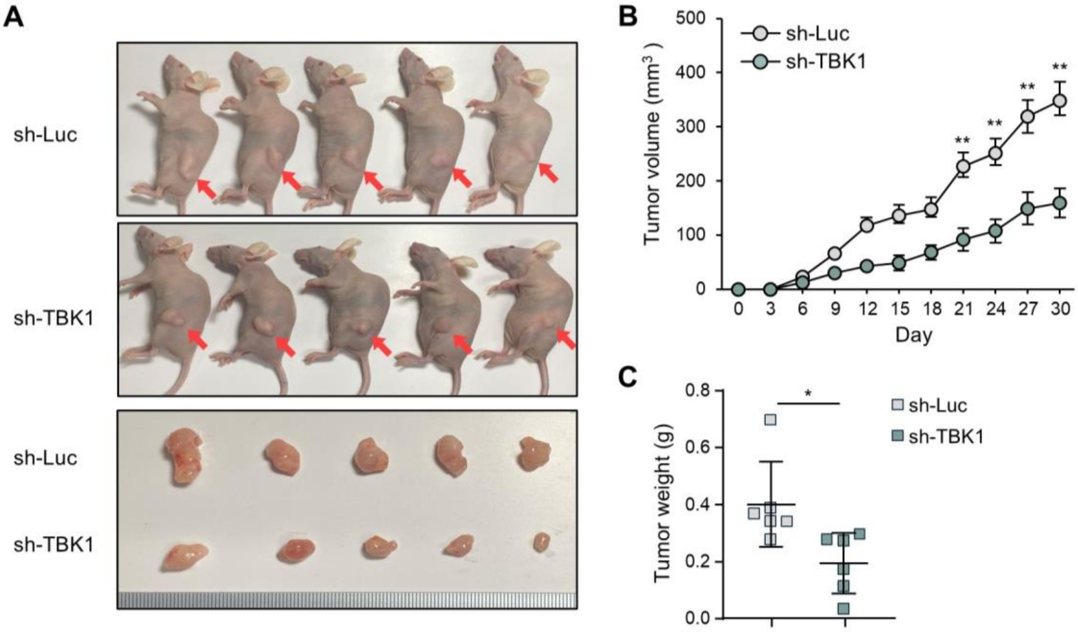

Figure S9. *TBK1* knockdown suppresses tumor growth *in vivo*. (A) Representative images of subcutaneous xenograft tumors in nude mice injected with lentiviral sh-TBK1- or sh-Luc-infected HEC-1A cells. (B) Tumor growth curve in each group. (C) Tumor weight in each group. Data are represented as the mean ± SEM. *p < 0.05 and **p < 0.01.
